# Arterial angioembolisation versus pre-peritoneal pelvic packing in haemodynamically unstable patients with complex pelvic fractures: a meta-analysis

**DOI:** 10.1007/s00068-023-02389-4

**Published:** 2023-11-14

**Authors:** Filippo Migliorini, Federico Cocconi, Inger Schipper, Kaj ten Duis, Ingo Marzi, Radko Komadina, Frank Hildebrand, Klaus Wendt

**Affiliations:** 1 Department of Orthopaedics, Trauma and Reconstructive Surgery, RWTH University Hospital, Pauwelsstraße 30, 52074 Aachen, Germany; 2 Department of Orthopaedic and Trauma Surgery, Academic Hospital of Bolzano (SABES-ASDAA), Teaching Hospital of the Paracelsus Medical University, 39100 Bolzano, Italy; 3 Department of Orthopaedic and Trauma Surgery, Leiden University Medical Center, Leiden, The Netherlands; 4 Department of Orthopaedic and Trauma Surgery, University Medical Center Groningen, Groningen, The Netherlands; 5 Department of Orthopaedic and Trauma Surgery, University Hospital Frankfurt, Goethe University, Frankfurt, Germany; 6 Department of Orthopaedic and Trauma Surgery, Medical Faculty, University of Ljubljana, Ljubljana, Slovenia

**Keywords:** Pelvic fractures, Haemorrhage, Pre-peritoneal pelvic packing, Angioembolisation

## Abstract

**Introduction:**

Angioembolisation (AE) and/or pre-peritoneal pelvic packing (PPP) may be necessary for patients with complex pelvic fractures who are haemodynamically unstable. However, it remains unclear whether AE or PPP should be performed as an initial intervention and ongoing debates exist. This meta-analysis aimed to compare AE versus PPP in haemodynamically unstable patients with acute pelvic fractures. The primary outcomes of interest were to compare in-hospital mortality rate and number of blood units transfused. Secondary outcomes included evaluating differences in the time from diagnosis to treatment, as well as the length of stay in the intensive care unit (ICU) and hospital.

**Methods:**

All clinically relevant studies comparing AE versus PPP in patients with complex pelvic fractures and haemodynamic instability were accessed. The 2020 PRISMA guidelines were followed. In September 2023, the following databases were accessed: PubMed, Web of Science, Google Scholar and Embase, without constraint.

**Results:**

Data from 320 patients were collected (AE: 174; PPP: 146). The mean age on admission was 47.4 ± 7.2 years. The mean Injury Severity Score (ISS) on admission was 43.5 + 5.4 points. Baseline comparability was observed in ISS (*P* = 0.5, Table 3) and mean age (*P* = 0.7, Table 3). No difference was reported in mortality rate (*P* = 0.2) or rate of blood units transfused (*P* = 0.3). AE had a longer mean time to the procedure of 44.6 min compared to PPP (*P* = 0.04). The mean length of ICU and hospital stay were similar in both groups.

**Conclusion:**

Despite the longer mean time from admission to the procedure, no significant differences were found between AE and PPP in terms of in-hospital mortality, blood units transfused, or length of ICU, and hospital stay. These findings should be interpreted considering the limitations of the present study. High-quality comparative research is strongly warranted.

**Level of evidence:**

Level IV, meta-analysis.

## Introduction

Complex pelvic fractures represent approximately 3% of all skeletal injuries and are associated with significant morbidity and mortality [1, 2]. In addition to peripelvic soft-tissue damage, pelvic fractures may involve the genitourinary system, lower gastrointestinal system, muscles, nerves, or blood vessels [3–7]. Following a proper treatment algorithm, the mortality rate in high-energy pelvic fractures dropped from 66.7% to 18.7% [8]. Haemorrhage is the leading cause of death, with a mortality rate reaching 35% [9, 10]. The management of haemodynamically unstable pelvic fractures is complex and requires a prompt, well-structured and multidisciplinary approach [11, 12]. The therapeutic algorithm must be individually tailored and adapted to the circumstances and characteristics of the admission centre [13–16]. If possible, an accident history and clinical examination should be obtained from each patient. It should be noted that pelvic instability should only be checked once during the clinical examination, so as not to worsen the injury or increase the haemorrhaging [17, 18]. The methods to ascertain the instability are debated. The validity of conventional radiographs in complex pelvic injuries is unclear; at the same time, the use of computer tomography, albeit useful, could cost important time. Newer types of CT scans located in the shock room could shorten this delay [19–22]. In haemodynamically unstable patients with complex pelvic fractures and free intra-abdominal fluid on FAST (focussed abdominal trauma-specific sonography), immediate exploratory laparotomy may be indicated [23–29]. External fixation may be performed following laparotomy. Stabilisation of the dorsal pelvic ring is a prerequisite for successful pelvic tamponade. In haemodynamically unstable patients with complex pelvic fractures without free intra-abdominal fluid, stabilisation is necessary at first, using a pelvic binder, anteriorly fixing the external fixator, or posteriorly fixing systems such as the pelvic clamp [30–32]. In persistent pelvic haemorrhage, the use of angioembolisation (AE) and/or pre-peritoneal pelvic packing (PPP) could be necessary. However, whether AE or PPP could be performed first is still unclear and debates are ongoing. The present meta-analysis was conducted to compare AE versus PPP in haemodynamically unstable patients with acute pelvic fractures. Primary outcomes of interest were in-hospital mortality and number of blood units transfused. Secondary outcomes of interest were the differences in time elapsed from diagnosis to treatment procedure and length of intensive care unit (ICU) and hospital stay.

## Methods

### Eligibility criteria

All clinical studies comparing AE versus PPP in patients with complex pelvic fractures and haemodynamic instability and published in peer-reviewed journals were eligible for assessment. Fitting with the author’s language capabilities, articles in English, German, Italian, French, and Spanish were considered. Only studies with levels I to IV of evidence, according to the Oxford Centre of Evidence-Based Medicine [33], were considered. Reviews, opinions, letters, and editorials were not considered. Animals, in vitro, biomechanics, computational, and cadaveric studies were not eligible. Missing quantitative data under the outcomes of interests warranted the exclusion of a study.

### Search strategy

This study was conducted according to the Preferred Reporting Items for Systematic Reviews and Meta-Analyses: the 2020 PRISMA statement [34]. The PICO algorithm was preliminarily established:

- P (Problem): Haemodynamically unstable patients with acute pelvic fractures
- I (Intervention): Pre-peritoneal pelvic packing
- C (Comparison): Arterial angioembolisation
- O (Outcomes): Mortality rate and blood units transfused, time elapsed from admission to treatment, length of ICU stay and hospitalisation.

In September 2023, PubMed, Web of Science, Google Scholar and Embase were accessed with no time constraints. The following matrix of keywords used in each database to accomplish the search was: pelvic OR pelvis AND fractures AND haemodynamic OR haemodynamically OR unstable OR instability OR bleeding OR haemorrhage AND peritoneal AND pelvic packing AND/OR arterial angioembolisation AND/OR versus AND survivorship OR mortality OR dead AND blood units AND transfused OR transfusion AND treatment OR management AND ICU OR intensive care unit OR hospitalisation OR outcome. The Boolean operator AND/OR was used for the database search. No additional filters were used in the database search.

### Selection and data collection

Two authors (F.M. and F.C.) independently performed the database search. All the resulting titles were screened by hand and, if suitable, the abstract was accessed. The full text of the abstracts matching the topic was accessed. If the full text was not accessible or available, the article was not considered for inclusion. A cross-reference of the bibliography of the full-text articles was also performed for inclusion. Disagreements were debated and solved by a third author (K.W.).

### Data items

Two authors (F.M. and F.C.) independently performed data extraction. The following data at baseline were extracted: author, year of publication and journal, country, study design, Injury Severity Score (ISS) [35], number of patients and related mean age. The primary outcomes of interest were to compare AE versus PPP in mortality rate and blood units transfused. Secondary outcomes of interest were to evaluate differences in time elapsed from diagnosis to treatment and length of ICU and hospital stay.

### Methodological quality assessment

The risk of bias was evaluated following the guidelines in the Cochrane Handbook for Systematic Reviews of Interventions [36]. The Risk of Bias in Nonrandomized Studies of Interventions (ROBINS-I) was used to assess the methodological quality assessment [37]. This scoring was conducted by two reviewers (F.M. and F.C.) independently. The quality of evidence of collective outcomes was evaluated using the Grading of Recommendations, Assessment, Development, and Evaluation (GRADE) system [38, 39].

### Synthesis methods

The statistical analyses were performed by the main author (F.M.) following the recommendations of the Cochrane Handbook for Systematic Reviews of Interventions [40]. IBM SPSS software version 25 was used for descriptive statistics and the assessment of baseline comparability. Mean and standard deviations were used. To assess baseline comparability the two-tailed unpaired *t* test was performed, with values of *P* > 0.05 considered satisfactory. To compare AE versus PPP, a meta-analysis was conducted using the software Review Manager 5.3 (The Nordic Cochrane Collaboration, Copenhagen, Denmark). For continuous data, the inverse variance method with mean difference (MD) effect measure was used. For binary data, the Mantel–Haenszel method with odds ratio (OR) effect measure was used. The CI was set at 95% in all the comparisons. Heterogeneity was evaluated through Higgins-*I*^2^ and *χ*^2^ tests. If *P*_*χ*2_ > 0.05, no statistically significant heterogeneity was found. If *P*_*χ*2_ < 0.05, the heterogeneity of the Higgins-*I*^2^ was evaluated. Values of Higgins-*I*^2^ > 60% indicate high heterogeneity. A fixed-effect model was set as default. When high heterogeneity was detected, a random model effect was used. Forest and funnel plots of each comparison were performed. Overall values of *P* < 0.05 were considered statistically significant.

## Results

### Study selection

**Fig. 1:**
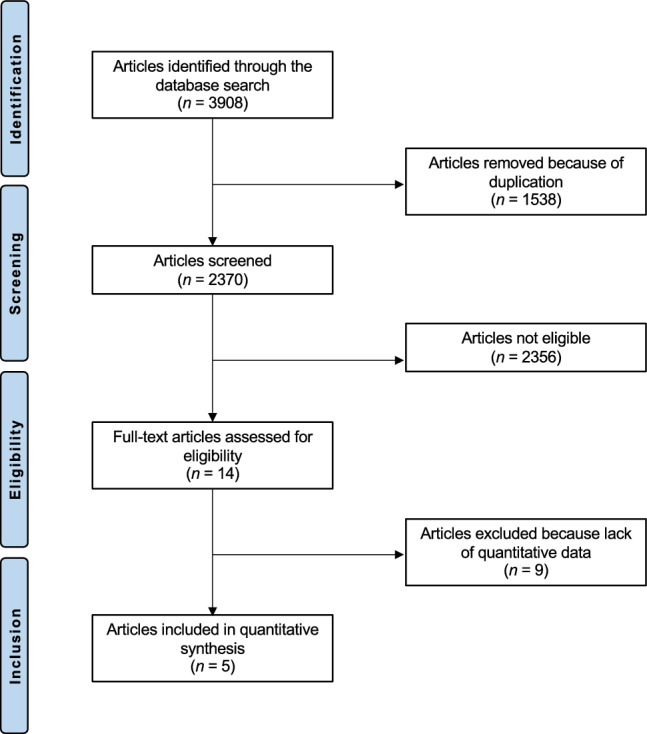
PRISMA flow chart of the literature search

The literature search resulted in 4285 articles. Of them, 1538 were excluded as they were duplicates. An additional 2356 studies were excluded for reasons of not matching the topic (*N = *2101), study design (*N = *193), combining several procedures (*N = *56), uncertain results (*N = *3) and language limitations (*N = *3). A further nine studies were excluded as they did not report quantitative data under the outcomes of interest. Finally, five studies were included in the present meta-analysis. The results of the literature search are shown in Fig. 1.

### Risk of bias assessment

The ROBINS-I was applied to investigate the risk of bias of non-RCTs. The overall risk of bias was moderate (Table 1).

**Table 1:** ROBINS-I of non-RCTs

Authors, year	Confounding	Selection of participants	Classification of interventions	Deviations from intervention	Missing data	Measurement of outcomes	Selection of reported results	Overall
Cheng et al., 2015 [41]	High	Low	High	Moderate	Moderate	Low	Low	Moderate
Kim et al. 2023 [42]	Moderate	Moderate	Moderate	High	High	High	High	Moderate
Li et al. 2016 [43]	Low	Low	Moderate	Moderate	Low	Low	Moderate	Moderate
Osborn et al. 2009 [44]	Moderate	Low	Low	Moderate	High	Moderate	Moderate	Moderate
Tai et al. 2011 [45]	Moderate	High	Moderate	Low	Moderate	High	Moderate	Moderate

### Study characteristics and results of individual studies

Data from 320 patients were collected (AE: 174; PPP: 146). The mean age on admission was 47.4 ± 7.2 years. The mean ISS on admission was 43.5 + 5.4 points. The generalities and demographics of the included studies are shown in Table 2.

**Table 2:** Study characteristics and patient group demographics

Authors, year	Journal	Country	Design	Definition of haemodynamic instability (at admission)	Group	ISS	Patients (*n*)	Mean age
Cheng et al. 2015 [41]	Emerg Med J	China	Prospective	SBP < 90 mm/Hg despite 2 L of crystalloids	AE	45	76	46.84
					PPP	40	49	45.37
Kim et al. 2023 [42]	Asian J Surg	South Korea	Retrospective	SBP < 90 mmHg and peripheral vasoconstriction, altered consciousness; and/or dyspnoea or (b) as SBP > 90 mmHg but requiring vasopressors; and/or base excess > 5 mmol/L and/or shock index > 1 and/or blood transfusion units of > 4/24 h	AE	34.8	38	56.7
					PPP	39.2	37	58.9
Li et al. 2016 [43]	Injury	China	Prospective	SBP < 90 mm Hg despite 4 U PRBCs	AE	43	27	40
					PPP	48	29	43
Osborn et al. 2009 [44]	Injury	USA	Prospective	SBP < 90 mm Hg despite 4 PRBCs	AE	45.9	20	39.5
					PPP	54.7	20	37.9
Tai et al. 2011 [45]	J Trauma	China	Retrospective	SBP < 90 mm Hg despite 2 L crystalloid	AE	42.3	13	44.8
					PPP	42	11	51.2

*RCT* randomised controlled trial; *ISS* Injury Severity Score; *SBP* systolic blood pressure; *PRBC* packed red blood cells

### Baseline comparability

On admission, ISS and mean age were comparable for both groups (Table 3).

**Table 3:** Baseline comparability

Endpoint	AE (*N = *174)	PPP (*N = *146)	MD	*P*
ISS	42.2 ± 4.4	44.8 ± 6.5	2.6	0.5
Mean age	45.6 ± 7.0	47.3 ± 8.1	1.4	0.7

*AE* angioembolisation; *PPP* peritoneal pelvic pack; *MD* mean difference; *ISS* Injury Severity Score

### Mortality rate

**Fig. 2:**
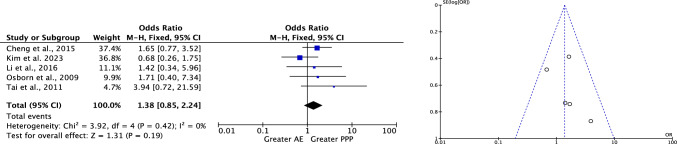
Meta-analysis of the comparison: rate of blood units transfused

No difference was reported in mortality rate (*P* = 0.2, Fig. 2).

### Blood units transfused

**Fig. 3:**
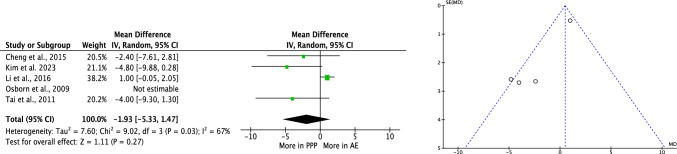
Meta-analysis of the comparison: rate of blood units transfused

No difference was reported in the transfused blood unit rate (*P* = 0.3, Fig. 3).

### Quality of the recommendations

**Fig. 4:**
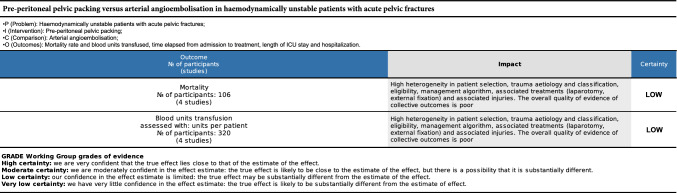
The overall quality of evidence of collective outcomes according to the GRADE approach was low

GRADE found a low quality of the recommendations (Fig. 4).

### The secondary outcome of interest

AE demonstrated a greater mean time to procedure of 44.6 min (*P* = 0.04) compared to PPP. The mean length of ICU and hospital stay were similar. These results are shown in Table 4.

**Table 4:** Overall results of the meta-analyses

Endpoints	Patients	95% CI	EE	Model	*I*^2^ (%)	*P*
Time to procedure	155	2.44–86.85	44.64	Random	78	0.04
ICU stay	96	1.65 to − 0.49	0.58	Fixed	0	0.3
Hospital stay	165	7.89 to − 11.38	1.74	Fixed	0	0.7

*CI* confidence interval; *EE* estimated effect; *ICU* intensive care unit

## Discussion

According to the main findings of the present study, AE and PPP in haemodynamically unstable patients with complex pelvic fractures are associated with similar in-hospital mortality rates and numbers of blood units transfused. No difference was found in the length of ICU stay or hospitalisation between AE and PPP. AE requires a longer time from admission to the procedure. PPP is performed without delay by the surgical team itself, but for AE interventional radiologists should be available.

Depending on the setting, the initial application of the pelvic binder (prehospital), pelvic clamp (emergency room) or external fixator (surgical theatre) in patients with unstable pelvic fractures and haemodynamic instability is recommended [46]. A prompt ventral external fixation is a fast procedure that leads to immobilisation of the pelvis and prevents further dislocation of the bony fragments but cannot exert sufficient dorsal compression. The pelvic clamp enables direct compression in the area of the dorsal pelvic ring and is, therefore, used to stabilise vertically unstable C injuries. However, to avoid iatrogenic injuries, such as ileal perforation or overcompression, especially in the case of lack of experience, its use has declined [47, 48].

Pelvic bleeding can be attributed to several sources: the presacral venous plexus, the pelvic bone itself and the pelvic arteries [49–52]. In the management of complex pelvic fractures with severe haemorrhage, haemostasis using PPP plays an important role. PPP only makes sense in connection with mechanical stabilisation, such as with a pelvic clamp or external fixator. This is especially true for the dorsal ring, as the stability provided to the anterior ring by an external fixator is less relevant for the effectiveness of PPP. In most cases, complex pelvic fractures are associated with venous bleeding, particularly from the presacral venous plexus, which can generally be controlled with PPP. The surgical access is achieved using an approximately 8-cm-long infra-umbilical longitudinal or transverse incision just above the pubis in the direction of the umbilicus; the linea alba is opened longitudinally. No preparation is necessary below the fascia, and the bladder can be lifted from the fractured site. Lap pads are inserted into the retroperitoneal space along the iliac vessels on each side. These are clamped between the posterior and anterior pelvic rings. Since the source of bleeding is the retroperitoneal part of the pelvis, no laparotomy is required. The duration of the procedure for an experienced surgeon should be about 20 min. Traditionally, every 24 or 48 h, the abdominal laparotomic pads shall be changed, which also allows a second-look surgery [44]. Following the acute care, further inpatient care takes place in the ICU and, if necessary, second- or third-look surgeries after 48 or 72 h [53, 54]. If the patient continues to bleed, additional AE should be considered.

In lesser cases, pelvic haemorrhages are of arterial origin [55, 56]. In the middle part of the superior and inferior pubic rami, along the ischial ramus, in the apex of the greater sciatic notch and the vicinity of the ventral part of the sacroiliac joint, lie the arteries which run close to the bone, and fractures in this location are most at risk of arterial haemorrhage [57–60]. Therefore, vertically displaced fractures in these areas are worthy of strict follow-ups. AE is an appropriate procedure to control arterial bleeding in patients with pelvic fractures; early AE within 3 h correlates significantly with better patient outcomes [3, 61–64]. AE should be performed in the event of haemodynamic instability and persistent bleeding despite pelvic stabilisation and the absence of significant alternative sources of bleeding. For a successful AE, an arterial blush of contrast media should be visible in the initial CT [65–67]. If suspicion of arterial bleeding persists after performing the AE, pelvic packing and stabilisation are warranted. If arterial bleeding persists, a repeated angiography and, if necessary, AE is indicated [68, 69].

Longer time from indication to procedure in patients who undergo AE compared to PPP was evidenced in the present meta-analysis. This finding concords with those reported by previous reports. Moreover, between-studies time to AE was highly heterogeneous, ranging from 45 to 130 min. Indeed, AE can only be performed in dedicated centres with specially trained staff [4, 70], whilst PPP can be quickly performed in the emergency department. Goldenshluger et al. [71] found no difference in patients who had undergone PPP in the emergency department versus the operating room in mortality, transfused blood units, surgical site infections or length of hospitalisation. Hauschild et al. [72] compared PPP versus PPP followed by AE (PPP combined with AE) in haemodynamically unstable patients with complex pelvic fractures: no patients (0 of 17) allocated to PPP combined with AE died from haemorrhage compared to 24% (32 of 135) who underwent PPP in isolation [72]. However, patients who underwent PPP combined with AE required a greater number of blood transfusions and showed a higher incidence of adult respiratory distress syndrome plus a tendency towards increased multiple organ failures [72]. It remains unclear whether these results are influenced by the greater arterial haemorrhages observed in patients who underwent PPP combined with AE or by the AE in isolation. On the other hand, Ming Hsu et al. [30] compared PPP combined with AE (*N = *14) versus AE in isolation (*N = *10): despite the combined group having a higher Injury Severity Score at admission, this group showed a lower rate of mortality and blood transfusion requirement compared to the AE-only group. Similar survivorship, blood units transfused, and length of ICU and hospital stay were evidenced between AE and PPP.

The present meta-analysis has several limitations. The retrospective design of most studies increases the risk of selection bias and negatively impacts the reliability of the present results. Moreover, the limited sample size and the number of included studies also have a negative impact on the validity of the present conclusions. The definition of haemodynamic instability was highly heterogeneous amongst the included studies, as was the time to admission to the emergency department. Data on blood pressure were not available in most studies. Between and within studies, different devices of external fixation (e.g. external fixator frame, pelvic C-clamp) were used. The therapeutic algorithm of haemodynamically unstable patients with complex pelvic fractures was highly heterogeneous and patients before haemorrhage control underwent different emergency advanced life-support protocols. Given the lack of information in most studies on the therapeutic algorithms used, subgroup comparisons were not conducted. Moreover, some authors included patients who had undergone laparotomy before pelvic haemorrhage control. The type of fractures included for analysis was often biassed and a formal classification was not used in most studies. Finally, the dynamics of the traumas, the presence of open or closed fractures and other associated injuries were seldom described and poorly standardised. Given these evident limitations, solid recommendations cannot be inferred. International consensus on the proper management algorithm is strongly required. The dynamics of the instability of patients with severe pelvic trauma are challenging to analyse in a meta-analytic fashion. This dynamic cannot be reflected by a single blood pressure value and leads to the risk of bias. If the patient is continuously unstable and not responding to resuscitation (non-responder), most surgeons would choose PPP, which is immediately available. However, if the unstable patient responds to resuscitation (transient responder), the interventional radiologist has time to wait until AE is possible. Therefore, haemodynamic instability and the response to resuscitation is a dynamic situation, leading to the optimal surgical/interventional treatment. Concluding, the choice between PPP and AE is complex and requires additional investigation. The management of haemodynamically unstable patients with complex pelvic fractures is challenging and debated. If the cause of instability is the complex pelvic fracture and devices of external fixation are not enough to control haemostasis, the surgeon should opt between PPP and AE. Formal recommendations cannot be inferred from this study design. The nature of the bleeding (venous and/or arterial), the surgeon experience, the availability of interventional radiologists and trained staff in a timely fashion, fracture type and trauma dynamics, comorbidities and patient characteristics must be considered. Internationally accepted protocols are strongly required to establish the most appropriate algorithm to manage haemodynamically unstable patients with complex pelvic fractures.

## Conclusion

Despite a longer mean time to procedure, there were no significant differences between AE and PPP in terms of in-hospital mortality rate, blood units transfused or length of stay in the ICU or hospital. These results must be interpreted in light of the limitations of the present study.

## Data Availability

The datasets generated and/or analysed during the current study are available throughout the manuscript.
